# Motor inhibition errors and interference suppression errors differ systematically on neural and behavioural features of response monitoring

**DOI:** 10.1038/s41598-024-66364-8

**Published:** 2024-07-10

**Authors:** Elisa Porth, André Mattes, Jutta Stahl

**Affiliations:** Department of Individual Differences and Psychological Assessment, University of Cologne, Pohligstraße 1, 50969 Cologne, Germany

**Keywords:** Cognitive neuroscience, Neuroscience, Psychology

## Abstract

Action inhibition and error commission are prominent in everyday life. Inhibition comprises at least two facets: motor inhibition and interference suppression. When motor inhibition fails, a strong response impulse cannot be inhibited. When interference suppression fails, we become distracted by irrelevant stimuli. We investigated the neural and behavioural similarities and differences between motor inhibition errors and interference suppression errors systematically from stimulus-onset to post-response adaptation. To enable a direct comparison between both error types, we developed a complex speeded choice task where we assessed the error types in two perceptually similar conditions. Comparing the error types along the processing stream showed that the P2, an early component in the event-related potential associated with sensory gating, is the first marker for differences between the two error types. Further error-specific variations were found for the parietal P3 (associated with context updating and attentional resource allocation), for the lateralized readiness potential (LRP, associated with primary motor cortex activity), and for the P_e_ (associated with error evidence accumulation). For motor inhibition errors, the P2, P3 and P_e_ tended to be enhanced compared to successful inhibition. The LRP for motor inhibition errors was marked by multiple small response impulses. For interference suppression errors, all components were more similar to those of successful inhibition. Together, these findings suggest that motor inhibition errors arise from a deficient early inhibitory process at the perceptual and motor level, and become more apparent than interference suppression errors, that arise from an impeded response selection process.

## Introduction

Action inhibition and error commission are inherent parts of everyday life. When inhibition fails, error processing is crucial to allow learning from the mistake and to reach the goal in the next attempt. It is important to consider that inhibition is not a unitary construct, but comprises at least two facets: interference suppression and motor inhibition^1,2^. When *interference suppression* is deficient, we become distracted by irrelevant stimuli and lose focus of the target (e.g. when a television in the background distracts us from reading a paper), whereas in deficient *motor inhibition* a strong response impulse cannot be inhibited and one fails to override a prepotent response tendency (e.g. when pressing the send button although we just realised the email attachment is missing). Studies on the processing of inhibition errors are often based on the investigation of either motor inhibition or interference suppression. Thus, one cannot rule out that the theoretical implications are restricted to a specific error type (i.e. errors evoked by weak interference suppression or by weak motor inhibition). Importantly, although there is evidence for a correlation of the neural error processing markers of motor inhibition and interference suppression, there might be vital differences in the processing of the two error types^3^. Identifying the similarities and differences of motor inhibition errors and interference suppression errors requires a systematic comparison of both error types from stimulus onset to post-response adaptation. We developed a complex choice task to contrast motor inhibition errors and interference suppression errors directly, with the goal to generate insights into the involved processes before (perception, attention, inhibition, response preparation) and after (detection processes and adaptive mechanisms) error commission.

### Neural and behavioural features of inhibition errors

Cognitive neuroscience methods such as the event-related potential (ERP) in combination with behavioural measures help to monitor the processing course from stimulus onset over error processing to adaptive mechanisms of the two inhibition errors. Early stimulus processing and attention-related processes are investigated by ERP components that occur within the first 200 ms after stimulus onset, such as the P1^4,5^, the N1^6^ and the P2^7^. Variations in higher order decision processes and response preparation are reflected in the N2, the P3 and the lateralised readiness potential (LRP;^8,9^). The frontocentral N2 amplitude signals the need for motor inhibition, interference suppression, and conflict monitoring^10–13^, whereas the subsequent frontocentral P3 amplitude is associated with evaluation of the inhibition outcome^11,14^. The P3 latency is often used as a marker for stimulus evaluation time^15^. The LRP is a helpful time-critical marker that splits the response time (RT) into the pre-decision time (i.e. from stimulus onset to onset of activity of the contralateral primary motor cortex) and motor processing (i.e. from activation of the primary motor cortex to onset of the overt motor response^16^). After response execution, action monitoring is reflected by the error negativity (N_e_ or ERN;^17,18^), which is associated with error processing and conflict monitoring^19^, and the error positivity (P_e_,^17^), which is discussed as an indicator of aware error processing and error evidence accumulation^20,21^. Both components are typically more pronounced for errors than for correct responses (N_c_ or CRN, P_c_;^17,18^). Post-response behaviour such as post-error slowing (PES;^22,23^) and post-error improvement of accuracy^24^ deliver insights into consequences of error commission on subsequent performance.

### Challenges in investigating inhibition errors

The conceptual differences of motor inhibition errors and interference suppression errors challenged a proper comparison of the processing time course. Interference suppression is usually induced by irrelevant stimuli (e.g., Eriksen-Flanker task;^25^) or stimulus features (e.g. Stroop task;^26^), and a response is executed on every trial independent of whether interference suppression was successful. In contrast, motor inhibition tasks provoke erroneous responses by lowering the motor threshold (i.e. increasing the tendency to respond), by increasing the speed of evidence accumulation, or a combination of both, and successful motor inhibition usually means that a motor response is not given, as it is the case in Go/NoGo tasks^27,28^ and Stop-Signal tasks^29^. The fact that on interference suppression trials two types of overt responses can occur (i.e., correct responses or errors), but only one type can occur in motor inhibition tasks (i.e., commission errors) challenges contrasting the two inhibition error types. For a proper error-related contrast, the motor inhibition errors lack their physically equivalent counterparts (i.e. overt correct responses on trials where motor inhibition is needed).

### Previous comparisons of interference suppression and motor inhibition

Some studies developed so-called hybrid tasks, where both prepotent response tendencies and stimulus interference were induced within the same trial or mixed within blocks^30–33^. The authors usually focus on successful inhibition processes but not on the processing of unsuccessful inhibition. Using a hybrid-task, Brydges et al.^31^ found that the N2 peaked later for successful interference suppression than for successful motor inhibition. This suggested a different temporal resolution for motor inhibition and interference suppression to the effect that motor inhibition occurs before interference suppression. In contrast, Vuillier et al.^33^ did not observe this difference. However, both studies found a more negative N2 for trials where only motor inhibition was needed. Studies using the LRP concluded from their findings that interference suppression is part of response selection, whereas motor inhibition is part of response execution^34–36^. For the present approach investigating variations in error processing of the two inhibition types, hybrid tasks are less useful to disentangle motor inhibition and interference suppression as motor inhibition and interference suppression conditions are often combined either within trials^30,33^ or mixed within blocks^35^. Although combining the conditions allows for an investigation of interaction effects between the two inhibition types, participants also need additional cognitive resources to switch between the different inhibitory mechanisms; and both situations lead to the inhibitory processes becoming less distinct.

### Objective of the present study

**Figure 1:**
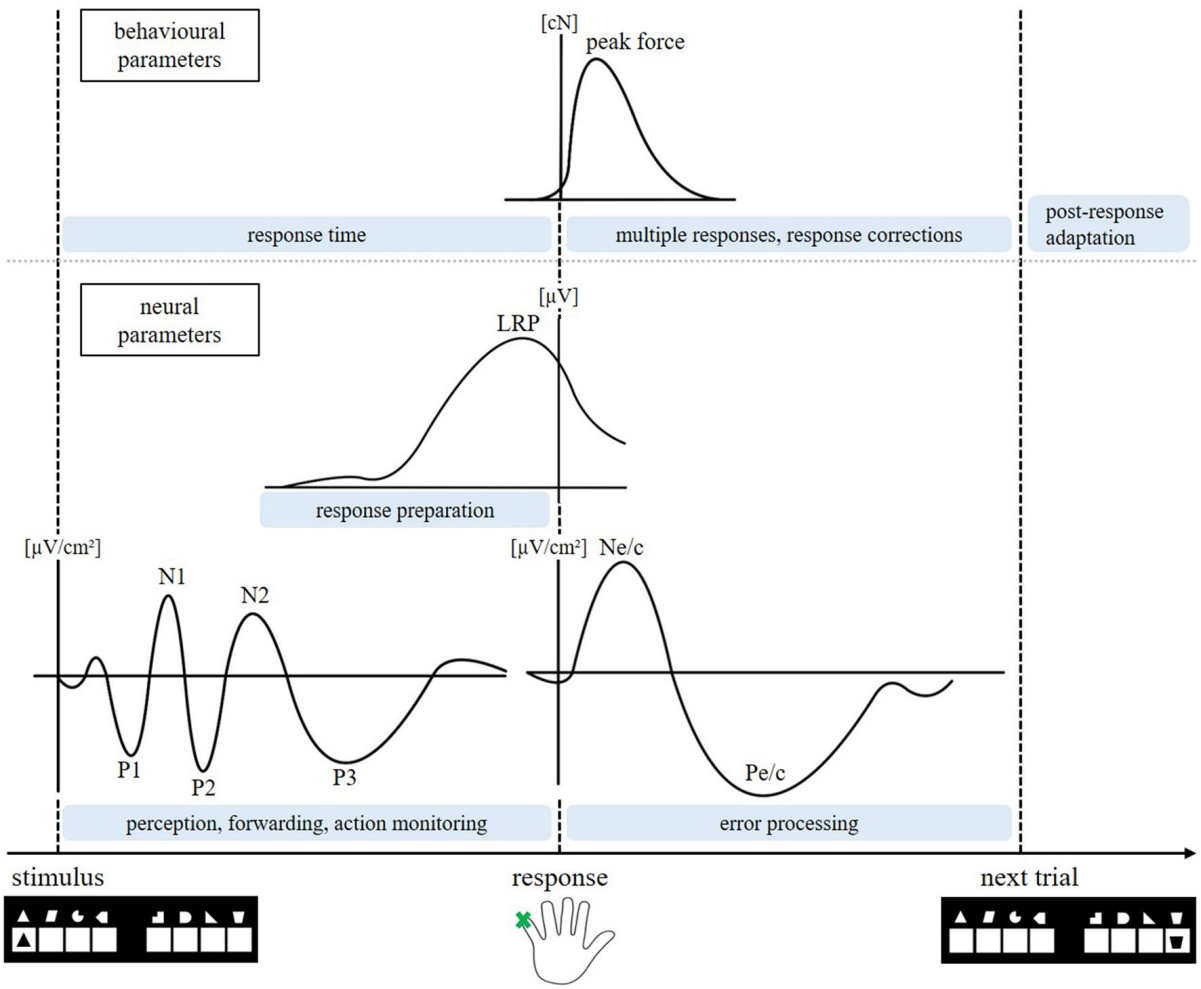
Processing stream from stimulus onset to post-response adaptation**.** *Note* Schematic overview on the investigated neural and behavioural correlates of the cognitive processing stream from stimulus onset over response execution to post-response behaviour.

The aim of the present study was to contrast motor inhibition errors and interference suppression errors directly and systematically in order to identify similarities and differences in cognitive processing between the two error types. To enable a direct comparison while overcoming previous task-related restrictions we implemented a modified version of the eight-alternative response task (8ART;^37,38^), which is a speeded choice task with a complex response selection. In the 8ART, we operationalized motor inhibition and interference suppression in two separate conditions and blocks, while using similar stimuli and the same instructions. In the interference suppression condition, we used distractor stimuli to evoke interference on the level of stimulus processing and response selection. We defined *interference suppression errors* as erroneous trials where participants responded to the distractor. We labelled the correct responses from this condition *interference suppression success*. In the motor inhibition condition, we manipulated the frequency of the congruency between the stimulus identity (target feature) and stimulus location (irrelevant feature). The more frequent, congruent trials had the purpose of inducing a prepotent response tendency towards stimulus location, which needed to be overridden during the less frequent, incongruent trials. We defined *motor inhibition errors* as trials where participants responded to the irrelevant stimulus location on the less frequent incongruent trials. We defined *motor inhibition success* as the correct responses from the less frequent incongruent trials, which form the counterpart to motor inhibition errors missing in typical tasks (e.g. Go/NoGo tasks). To gain a better conceptual understanding of motor inhibition errors and interference suppression errors, we contrasted the antecedences and consequences of both error types, starting with stimulus processing and ending with post-response adaptation. The cognitive processing stream and the respective neural and behavioural parameters are depicted in Fig. 1.

On a neural level, correlates of early perceptual stimulus processing (reflected by the P1 and N1) form the beginning of the processing stream and serve as potential markers of the dissociations between the two error types. The perceptual information is forwarded (a process often associated with the P2) to higher processing mechanisms such as action/conflict monitoring and conflict evaluation (associated with the N2 and P3). Meanwhile, the timing and course of preparatory patterns can be observed in the LRP. After response execution, error processing mechanisms are reflected by the N_e_ and P_e_. On the behavioural level, response times capture the process from stimulus onset to response execution. During the time window of the response, response force and the percentage of multiple responses are often interpreted as error-specific indicators for uncertainty and online inhibition^37,38^. As measures of post-error performance we assessed PES and post-response accuracy which often display modulations specific to different error types^39^.

The behavioural and neural parameters we assessed in the current study have been equally related to motor inhibition and interference suppression in the past. Studies that jointly investigated motor inhibition and interference suppression predominantly focused on successful inhibition and implemented hybrid tasks, which complicate a clear distinction between the two inhibition types. Considering the inconsistency of methodological approaches in the literature and the novelty of our task, we did not make predictions on potential differences between motor inhibition errors and interference suppression errors. Hence, our study was exploratory and served the purpose of generating first insights into these potential differences.

## Results

### Behavioural results

**Figure 2:**
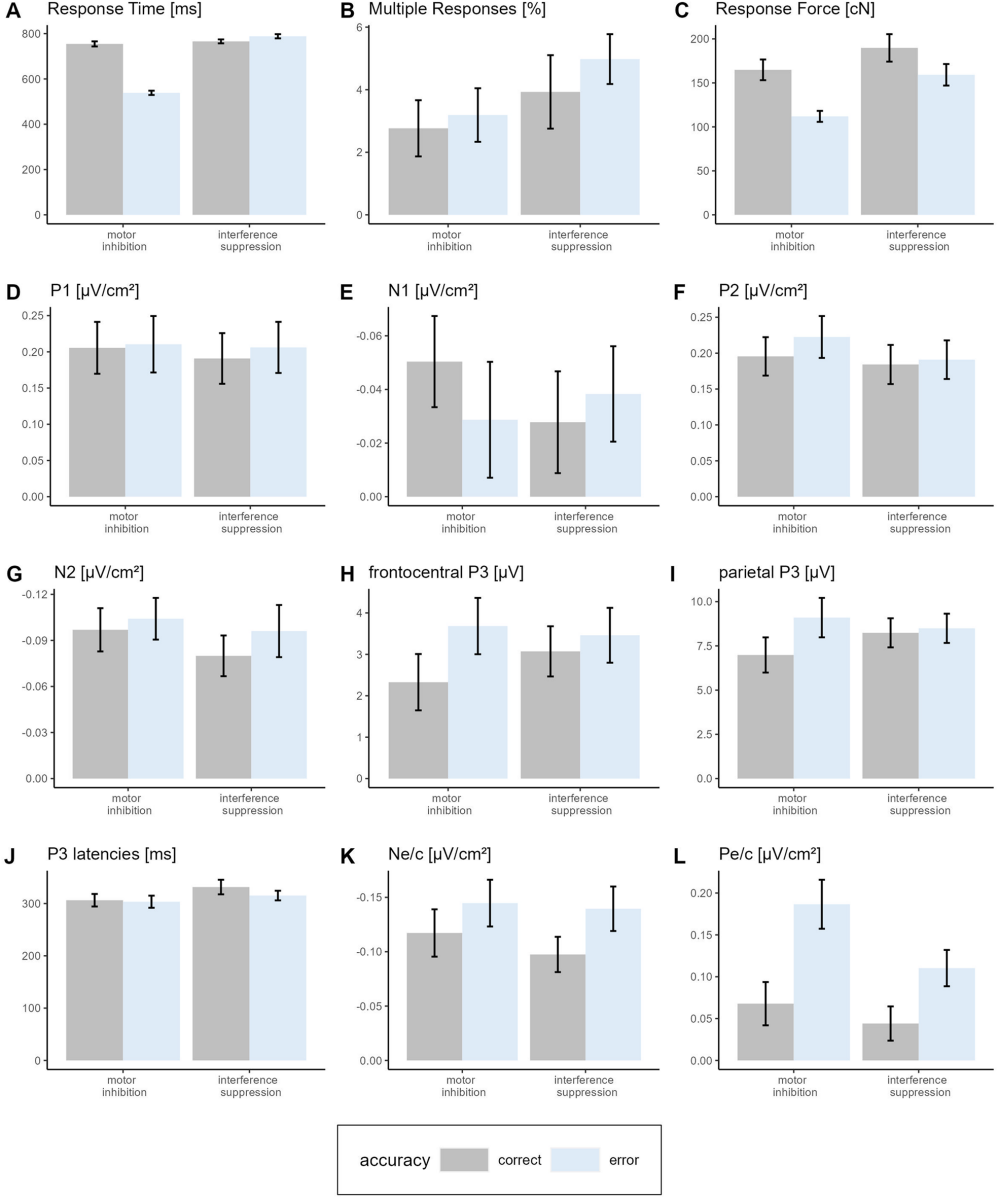
Descriptive statistics of behavioural and electrophysiological parameters. *Note* Descriptive statistics of the investigated neural and behavioural correlates separately for inhibition type (motor inhibition, interference suppression) and accuracy (correct, error).

After exclusions, the final sample comprised data of 30 participants. The descriptive statistics for the behavioural parameters are presented in Table 1 and Fig. 2A–C separately for each response type. In the following, we present the results of a 2 (Inhibition Type: motor inhibition vs. interference suppression) × 2 (Accuracy: success vs. error) repeated measures ANOVA for the behavioural parameters. In case of a significant interaction effect, we computed Tukey-adjusted post-hoc tests to explore the nature of the interaction.

**Table 1:** Means and standard errors of mean for the behavioural parameters of each inhibition type (motor inhibition, interference suppression) and accuracy (success, errors).

	Motor inhibition				Interference suppression			
	Success		Error		Success		Error	
	*M*	*SE*	*M*	*SE*	*M*	*SE*	*M*	*SE*
Response rates [%]	73.3	3.1	26.7	3.1	81.9	1.6	18.1	1.6
Response time [ms]	754.6	11.2	538.7	9.5	765.8	8.7	788.3	9.3
Multiple responses [%]	2.8	0.9	3.2	0.9	3.9	1.2	5.0	0.8
Response force [cN]	164.9	11.8	111.9	6.3	189.8	15.6	159.2	12.2
PES [ms]	–	–	11.5	6.3	–	–	29.4	5.4
Pre-error speeding [ms]	–	–	− 18.0	5.0	–	–	− 7.3	4.4
Post-response accuracy [%]	93.9	0.8	92.6	1.5	59.3	2.8	57.8	3.2

The computation of response rates is based solely on the trials included in the analyses. For response rates of the excluded trials see Fig. 7.

The ANOVA for RT revealed a significant main effect of Accuracy, *F*(1, 29) = 260.02, *p* < 0.001, η_p_^2^ = 0.90, of Inhibition Type, *F*(1, 29) = 572.56, *p* < 0.001, η_p_^2^ = 0.95, and of the interaction of Accuracy and Inhibition Type, *F*(1, 29) = 432.23, *p* < 0.001, η_p_^2^ = 0.94. Post hoc tests indicated that motor inhibition errors had the shortest RTs, all *p* values < 0.001, followed by motor inhibition success and interference suppression success, which did not differ significantly from each other, *p* = 0.529. Interference suppression errors had the longest RTs, all *p* values < 0.006.

For the percentage of multiple responses there was no significant main effect of Accuracy, *F*(1, 29) = 0.73, *p* = 0.401, η_p_^2^ = 0.02, but there was a significant main effect of Inhibition Type, *F*(1, 29) = 24.15, *p* < 0.001, η_p_^2^ = 0.45, indicating more multiple responses in the interference suppression condition (8.9 ± 1.7%) compared to the motor inhibition condition (6.0 ± 1.6%). The interaction of Accuracy and Inhibition Type was not significant, *F*(1, 29) = 0.42, *p* = 0.521, η_p_^2^ = 0.01.

There were more response corrections for interference suppression errors (3.2 ± 0.6%) than for motor inhibition errors (1.3 ± 0.4%), *t*(29) = − 2.92, *p* = 0.007, *d* = − 0.53.

For response force, there was a significant main effect of Accuracy, *F*(1, 29) = 52.53, *p* < 0.001, η_p_^2^ = 0.64, a significant main effect of Inhibition Type, *F*(1, 29) = 20.82, *p* < 0.001, η_p_^2^ = 0.42, and a significant interaction of Accuracy and Inhibition Type, *F*(1, 29) = 8.55, *p* = 0.007, η_p_^2^ = 0.23. Response force was highest for interference suppression success, all *p* values < 0.022, and lowest for motor inhibition errors, all *p* values < 0.001. Motor inhibition success and interference suppression errors did not differ significantly, *p* = 0.869.

PES was shown for motor inhibition errors (11.5 ± 6.3 ms), *t*(29) = 1.82, *p* = 0.039, *d* = 0.33, and interference suppression errors (29.4 ± 5.4 ms), *t*(29) = 5.49, *p* < 0.001, *d* = 1.00, and both error types differed significantly from each other, *t*(29) = 2.32, *p* = 0.027, *d* = 0.42. Pre-error speeding was shown for motor inhibition errors (-18.0 ms ± 5.0), *t*(29) = 3.63, *p* < 0.001, *d* = 0.66, and also, as a (non-significant) tendency, for interference suppression errors (-7.3 ms ± 4.4), *t*(29) = 1.65, *p* = 0.055, *d* = 0.30, and both error types differed significantly, *t*(29) = 2.53, *p* = 0.017, *d* = 0.46.

A post-response improvement of accuracy was neither shown in the motor inhibition condition, *t*(29) = 0.91, *p* = 0.370, *d* = 0.17, nor in the interference suppression condition, *t*(29) = 1.08, *p* = 0.291, *d* = 0.20.

### Electrophysiological results

The descriptive statistics for the electrophysiological parameters are presented in Table 2 and Fig. 2D–L. In the following, we present the results of a 2 (Inhibition Type: motor inhibition vs. interference suppression) × 2 (Accuracy: success vs. error) repeated measures ANOVA for the electrophysiological parameters. For the N2 and P3, a minimum number of 20 and 14 trials respectively is recommended to ensure internal consistency^40^. The analyses of the N_e/c_ and P_e/c_ should contain at least six error trials^41^. On average, 30 motor inhibition errors (*SD* = 17, *min* = 9, *max* = 74) and 49 interference suppression errors (*SD* = 27, *min* = 12, *max* = 115) were included in the ERP analyses (here: response-locked) after artefact rejection, indicating sufficient stability of the ERP measures.

**Table 2:** Means and standard errors of mean for electrophysiological parameters of each inhibition type (motor inhibition, interference suppression) and accuracy (success, errors).

	Motor inhibition				Interference suppression			
	Success		Error		Success		Error	
	*M*	*SE*	*M*	*SE*	*M*	*SE*	*M*	*SE*
P1 [µV/cm^2^]	0.21	0.04	0.21	0.04	0.19	0.03	0.21	0.04
N1 [µV/cm^2^]	− 0.05	0.02	− 0.03	0.02	− 0.03	0.02	− 0.04	0.02
P2 [µV/cm^2^]	0.20	0.03	0.22	0.03	0.18	0.03	0.19	0.03
N2 [µV/cm^2^]	− 0.10	0.01	− 0.10	0.01	− 0.08	0.01	− 0.10	0.02
Frontal P3 [µV]	2.3	0.7	3.7	0.7	3.1	0.6	3.5	0.7
Parietal P3 [µV]	7.0	1.0	9.1	1.1	8.2	0.8	8.5	0.8
P3 latency [ms]	306.3	12.0	303.5	11.9	331.5	13.9	315.3	9.2
S-LRP onset [ms]	502.8	18.3	672.1	5.9	582.0	16.6	394.7	11.0
R-LRP onset [ms]	− 140.2	9.6	− 223.8	3.8	− 114.3	7.1	− 139.3	25.0
N_e/c_ [µV/cm^2^]	− 0.12	0.02	− 0.14	0.02	− 0.10	0.02	− 0.14	0.02
P_e/c_ [µV/cm^2^]	0.07	0.03	0.19	0.03	0.04	0.02	0.11	0.02

S-LRP = stimulus-locked lateralised readiness potential. R-LRP = response-locked lateralised readiness potential.

### Early stimulus encoding: P1, N1 and P2

The ANOVA for the P1 amplitude showed no significant main effect of Accuracy, *F*(1, 29) = 3.35, *p* = 0.077, η_p_^2^ = 0.10, no significant main effect of Inhibition Type, *F*(1, 29) = 1.21, *p* = 0.280, η_p_^2^ = 0.04, and no significant interaction of Accuracy and Inhibition Type interaction*, F*(1, 29) = 0.43, *p* = 0.518, η_p_^2^ = 0.01.

For the N1 amplitude, there was no significant main effect of Accuracy, *F*(1, 29) = 0.55, *p* = 0.464, η_p_^2^ = 0.02, or Inhibition Type, *F*(1, 29) = 0.27, *p* = 0.604, η_p_^2^ < 0.01, but a significant interaction of Accuracy and Inhibition Type*, F*(1, 29) = 6.35, *p* = 0.018, η_p_^2^ = 0.18. However, post-hoc tests did not reveal any significant differences between the response types, all *p* values > 0.234.

For the P2 amplitude, there was a significant main effect of Accuracy, *F*(1, 29) = 6.40, *p* = 0.017, η_p_^2^ = 0.18, and of Inhibition Type, *F*(1, 29) = 5.53, *p* = 0.026, η_p_^2^ = 0.16, and a (non-significant) tendency for an interaction of Accuracy and Inhibition Type, *F*(1, 29) = 3.85, *p* = 0.059, η_p_^2^ = 0.12. Although this effect does not reach a 5% significance level, we cannot rule out that it was driven by a higher P2 amplitude for motor inhibition errors than for the other three response types (see descriptive statistics in Table 2).

### Inhibition and conflict monitoring: N2 and P3

For the N2 amplitude, there was no significant main effect of Accuracy, *F*(1, 29) = 3.03, *p* = 0.092, η_p_^2^ = 0.09, or of Inhibition Type, *F*(1, 29) = 1.68, *p* = 0.205, η_p_^2^ = 0.05, and no significant Accuracy × Inhibition Type interaction, *F*(1, 29) = 0.78, *p* = 0.384, η_p_^2^ = 0.03.

For the frontocentral P3 amplitude, there was a (non-significant) tendency for a main effect of Accuracy, *F*(1, 29) = 3.78, *p* = 0.062, η_p_^2^ = 0.12, there was no significant main effect of Inhibition Type, *F*(1, 29) = 0.53, *p* = 0.471, η_p_^2^ = 0.02, and there was a (non-significant) tendency for an interaction of Accuracy and Inhibition Type, *F*(1, 29) = 3.73, *p* = 0.063, η_p_^2^ = 0.11. Although this effect does not reach a 5% significance level, we cannot rule out that it was driven by a higher accuracy effect in the motor inhibition condition compared to the interference suppression condition (see descriptive statistics in Table 2). The topographies show that the positive activity in the time window of the P3 increases along the midline from frontocentral to parietal sites. For the parietal P3 amplitude (measured at Pz), there was a significant main effect of Accuracy, *F*(1, 29) = 7.69, *p* = 0.010, η_p_^2^ = 0.21. There was no significant main effect of Inhibition Type, *F*(1, 29) = 0.54, *p* = 0.468, η_p_^2^ = 0.02, but there was a significant interaction of Accuracy and Inhibition Type, *F*(1, 29) = 10.53, *p* = 0.003, η_p_^2^ = 0.27. Post hoc tests revealed that the parietal P3 amplitude for motor inhibition success was significantly lower than for motor inhibition errors, *p* = 0.011, significantly lower than for interference suppression success, *p* = 0.028, and tended to be lower than for interference suppression errors, *p* = 0.080. The other response types did not differ significantly, all *p* values > 0.524. In addition, the topographies showed a strong positive activity at occipital sites. The occipital positivity persisted over the entire trial course. Thus, in the time window of the P3 there might be overlapping activities from occipital sites and the P3 (Fig. 1 of the Supplementary Information).

The P3 latencies did not vary significantly with Accuracy, *F*(1, 29) = 1.12, *p* = 0.299, η_p_^2^ = 0.04, but there was a significant main effect of Inhibition Type, *F*(1, 29) = 4.21, *p* = 0.049, η_p_^2^ = 0.13, indicating longer latencies in the interference suppression condition. There was no significant interaction of Accuracy and Inhibition Type, *F*(1, 29) = 0.40, *p* = 0.532, η_p_^2^ = 0.01.

**Figure 3:**
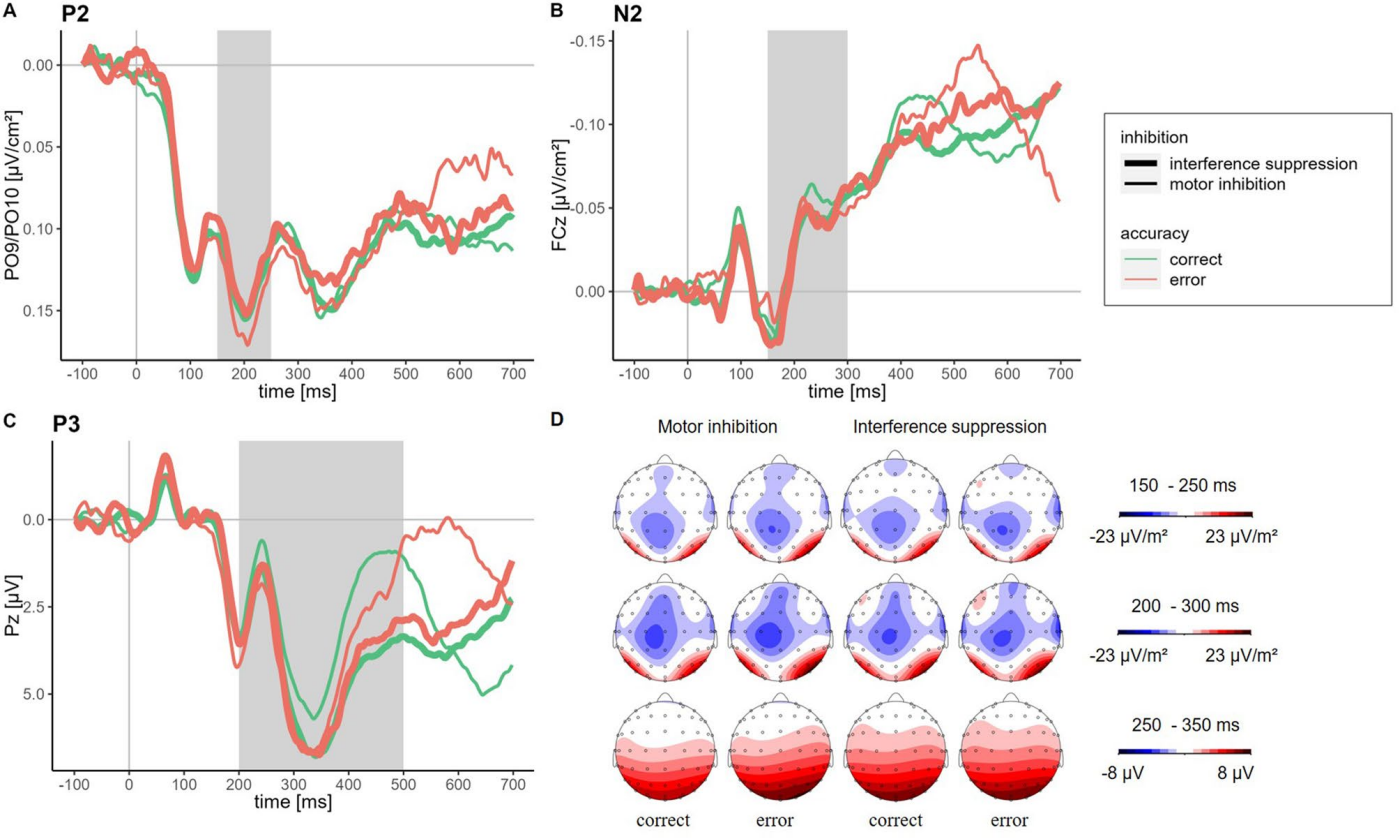
Averaged stimulus-locked ERP waveforms and topographical maps. *Note* Averaged current source density transformed waveforms (low-pass filtered at 40 Hz for illustration purposes) separately for inhibition type (thick lines: interference suppression, thin lines: motor inhibition) and accuracy (green lines: correct responses, red lines: errors) (**A**) of the P2, measured at PO9 and PO10 as the mean amplitude ± 2 data points around the positive peak in the time window of 150–250 ms after stimulus onset; (**B**) of the N2, measured at FCz as the mean amplitude ± 2 data points around the negative peak in the time window of 150–300 ms after stimulus onset. (**C**) Averaged untransformed waveforms (low-pass filtered at 40 Hz for illustration purposes) of the parietal P3, measured at Pz as the mean amplitude ± 2 data points around the positive peak in the time window of 200–500 ms after stimulus onset. (D) Topographical maps of the time windows around the components’ peaks.

The averaged ERP waveforms and the respective topographical maps of the P2, N2 and P3 are depicted in Fig. 3 (Figures for paired differences of the waveforms for the P2, N2, and P3 can be found in the Supplementary Information).

### From stimulus encoding to motor execution: LRP

For the LRP analyses, we included datasets from 24 of 30 participants because the analyses required a minimum number of six trials *per hand*, which led to the exclusion of several participants due to an insufficient number of error trials.

The ANOVA for the stimulus-locked LRP onset showed no significant main effect of Accuracy, *F*(1, 23) = 0.99, *p* = 0.330, η_p_^2^ = 0.04, but a significant main effect of Inhibition Type, *F*(1, 23) = 94.15, *p* < 0.001, η_p_^2^ = 0.80, and a significant interaction of Accuracy and Inhibition Type, *F*(1, 23) = 330.47, *p* < 0.001, η_p_^2^ = 0.93. Post hoc tests showed that the stimulus-locked LRP onset differed significantly between all response types, all *p* values < 0.001, with interference suppression errors having the earliest onset, followed by motor inhibition success, interference suppression success and then motor inhibition errors.

The ANOVA for the response-locked LRP onset showed a significant main effect of Accuracy, *F*(1, 23) = 14.58, *p* < 0.001, η_p_^2^ = 0.39, and Inhibition Type, *F*(1, 23) = 12.34, *p* = 0.002, η_p_^2^ = 0.35, and a significant interaction of Accuracy and Inhibition Type, *F*(1, 23) = 5.78, *p* = 0.025, η_p_^2^ = 0.20. Post-hoc tests showed that the response-locked LRP onset was significantly earlier for motor inhibition errors than for the other three response types, all *p* values < 0.019, which did not differ from each other, all *p* values > 0.071.

**Figure 4:**
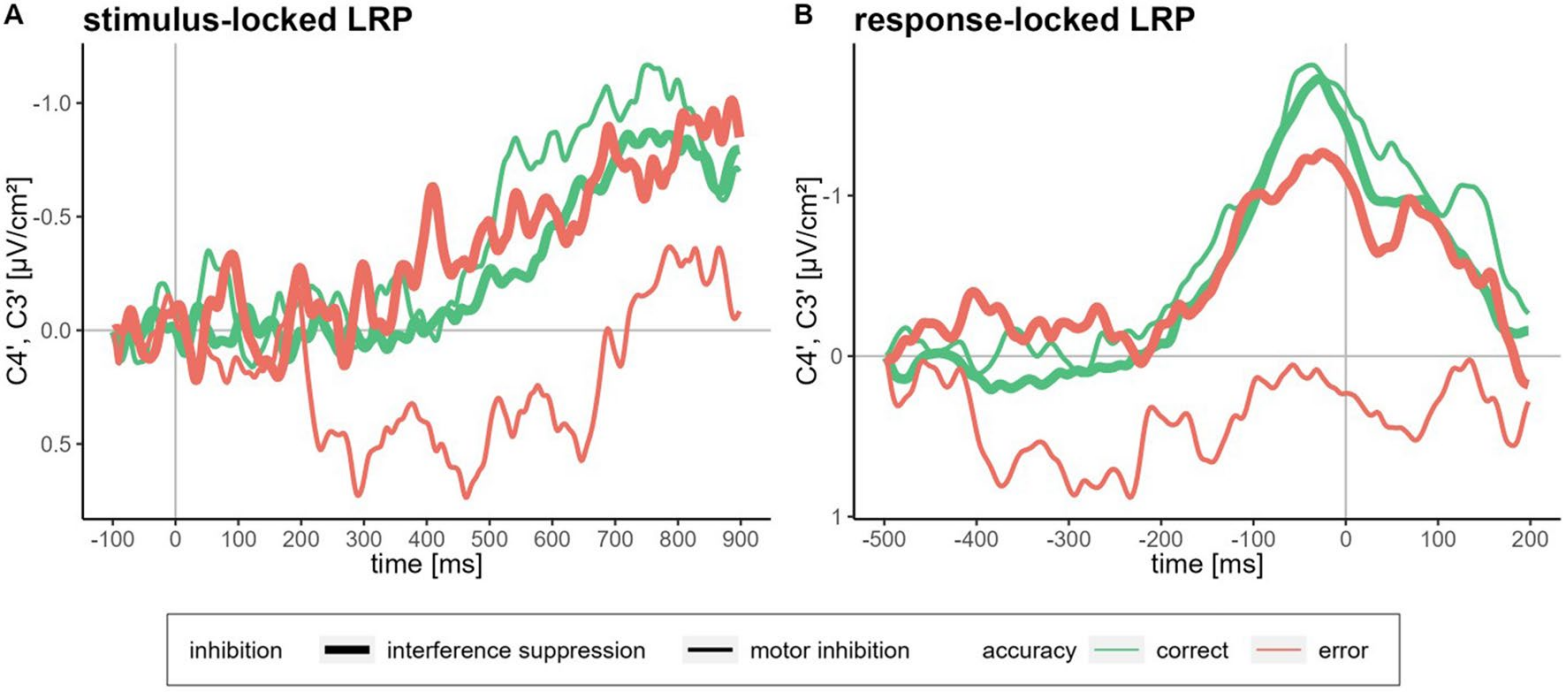
Averaged LRP waveforms**.** *Note* Averaged lateralised readiness potential (LRP) waveforms (low-pass filtered at 40 Hz for illustration purposes) derived from C3' and C4' electrode sites separately for inhibition type (thick lines: interference suppression, thin lines: motor inhibition) and accuracy (green lines: correct responses, red lines: errors) (**A**) stimulus-locked in the time window of − 100–900 ms; (**B**) response-locked in the time window of − 500–200 ms. We based LRP computations for errors on the hands with which the responses were executed.

The stimulus-locked and response-locked averaged LRP waveforms are depicted in Fig. 4 (paired differences of the LRP onsets are depicted in the Supplementary Information).

### Error processing: N_e/c_ and P_e/c_

For the N_e/c_ amplitude, there was a significant main effect of Accuracy, *F*(1, 29) = 7.63, *p* = 0.010, η_p_^2^ = 0.21, indicating higher amplitudes for errors than for successful responses. There was no significant main effect of Inhibition Type, *F*(1, 29) = 1.33, *p* = 0.259, η_p_^2^ = 0.04, and no significant interaction of Accuracy and Inhibition Type, *F*(1, 29) = 0.52, *p* = 0.478, η_p_^2^ = 0.02.

For the P_e/c_ amplitude there was a significant main effect of Accuracy, *F*(1, 29) = 16.19, *p* < 0.001, η_p_^2^ = 0.36, indicating higher amplitudes for errors than for successful responses, and a significant main effect of Inhibition Type, *F*(1, 29) = 10.47, *p* = 0.003, η_p_^2^ = 0.27, indicating higher amplitudes in the motor inhibition condition than in the interference suppression condition. There was a (non-significant) tendency for an interaction of Accuracy and Inhibition Type, *F*(1, 29) = 3.91, *p* = 0.057, η_p_^2^ = 0.12. Although this effect does not reach a 5% significance level, we cannot rule out that it was driven by the higher P_e_ amplitude for motor inhibition errors (see descriptive statistics in Table 2).

**Figure 5:**
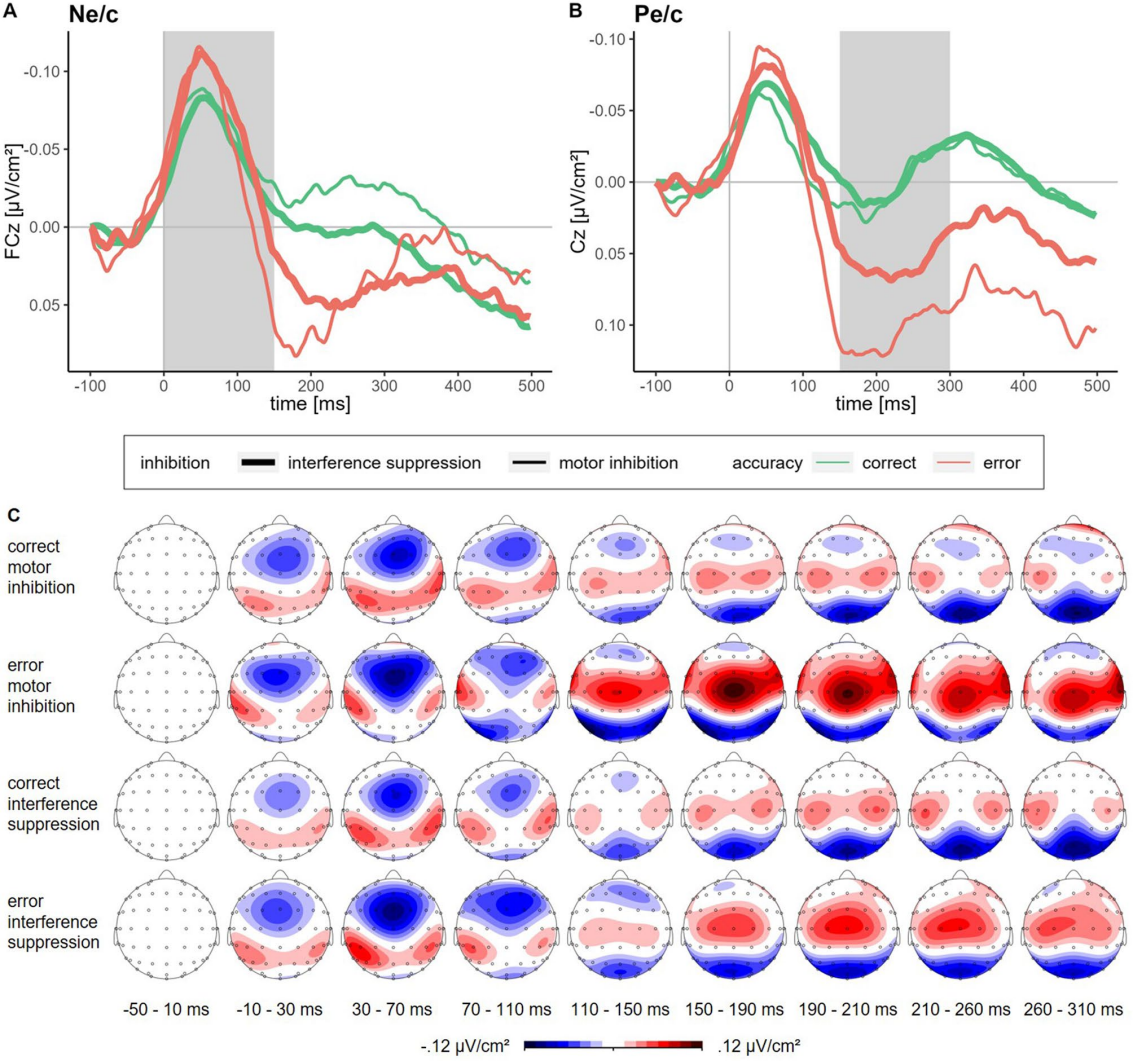
Averaged response-locked ERP waveforms and topographical maps**.** *Note* Averaged current source density transformed waveforms (low-pass filtered at 40 Hz for illustration purposes) separately for inhibition type (thick lines: interference suppression, thin lines: motor inhibition) and accuracy (green lines: correct responses, red lines: errors) (**A**) for the error negativity, measured at the FCz as the mean amplitude ± 2 data points around the negative peak in the time window of 0–150 ms after response onset; (**B**) for the error positivity, measured at the Cz as the mean amplitude ± 2 data points around the positive peak in the time window of 150–300 ms after response onset; (**C**) and the respective topographical maps.

The CSD-transformed averaged ERP waveforms for the N_e/c_ and P_e/c_, together with the topographical maps, are depicted in Fig. 5 (Figures for paired differences of the waveforms for the N_e/c_ and P_e/c_ can be found in the Supplementary Information).

## Discussion

We modified the 8ART^37,38^ to evoke motor inhibition and interference suppression in two perceptually similar conditions. We kept the perceptual differences between the two inhibition conditions as small as possible by using similar stimuli and instructions and reduced differences in motor execution by requiring a motor response on every trial. This was necessary to disentangle the response types psychologically rather than physically for the analyses following the Hillyard principle^42^. The modified 8ART unites different inhibitory concepts from traditional inhibition paradigms, which we will discuss in the following.

### Interference suppression and motor inhibition in the 8ART

In the interference suppression condition, we introduced distractors by mirroring the stimuli regarding their spatial orientation. Similar to traditional Flanker tasks, participants had to ignore the interference caused by these distractors and respond to the target stimulus instead. However, the distractors in our task were not independent of the task goal. A stimulus that can cause interference on one trial can become the correct stimulus on another trial. Moreover, distractors were always present in our task, while traditional Flanker tasks often contain congruent flanker stimuli with no distracting influence as well. Nevertheless, our paradigm implies similar types of conflict. As in traditional Flanker tasks, it creates conflict between target stimuli and distractor stimuli, as well as motor conflict between the associated responses.

In the motor inhibition condition, we introduced prepotent response tendencies towards target location by presenting the targets more frequently at response location. Similar to traditional Go/NoGo tasks, participants are instructed to inhibit the prepotent response tendency on some trials, and the information they need to identify these trials is available with stimulus onset. However, in the 8ART, successful stopping is accompanied by the selection of an alternative response^37,38^. This addition can also be found in stop-change paradigms, in which an additional signal indicates that an originally planned response must be inhibited, and an alternative response must be executed instead^43^. Stop-change tasks comprise three different processes: the first go process (i.e. responding according to a prepotent response tendency), a stop process (which cancels the first go response based on a signal), and a second go process (i.e. the execution of an alternative response)^44^. The stop process and the second go process might either occur serially or in a parallel, capacity-sharing manner^44^. Similar processes are relevant in the 8ART, even though there is no additional signal. The strategies that participants can apply to detect whether or not a response according to target location needs to be inhibited in the 8ART are similar to the strategies applied in the ignore signals task. The ignore signals task^45,46^ is a binary response task at which participants are presented with a stimulus and respond by selecting one of two response keys. On some trials, an additional auditory signal either indicates stopping and the suppression of motor execution or to proceed with response execution. Participants can apply similar strategies for successful responding in the ignore signals task and the 8ART: they can either *discriminate then stop* (i.e., in the 8ART they can first discriminate whether target and response location match and then inhibit a response towards target location if necessary; in the ignore signals task they can discriminate whether the auditory signal indicates stopping or response execution) or *stop then discriminate* (i.e. always inhibit a response towards target location or the auditory signal, respectively, and then proceed if no inhibition is indicated). In the 8ART, the first strategy is more efficient, as the congruent trials are more frequent than the incongruent trials. The latter strategy might be more suitable for less complex tasks which focus on the speed of stopping^47^.

Conflating different concepts of inhibition in the 8ART allowed us to compare motor inhibition errors and interference suppression errors directly and systematically, from stimulus onset to post-response adaptation. The results deliver interesting insights into the cognitive processing stream of the two error types, which we will discuss in the following.

### Early stimulus encoding

Starting our comparison at stimulus onset, the two inhibition errors showed no significant differences regarding the N1 and the P1, reflecting early attention-related perceptual encoding mechanisms^4^. The two inhibition types started to differ about 200 ms after the stimulus onset, demonstrated by the significantly enhanced P2 amplitude in the motor inhibition condition compared to the interference suppression. The P2 is assumed to reflect an early inhibitory mechanism that prevents irrelevant sensory features (e.g. colour, shape) from being processed further to protect the system from initiating erroneous actions^30^. Usually, a decreased P2 amplitude is associated with the success of this protective mechanism^30^. For motor inhibition errors, there was a (non-significant) tendency for an increased P2 amplitude compared to successful responses indicating a slight impediment of the protective mechanism. The irrelevant stimulus information (here, stimulus location) might not have been inhibited but processed further and was, in the end, incorrectly responded to. For interference suppression errors, no such tendency compared to successful responses was shown.

### Inhibition and conflict monitoring

The N2, which is assumed to reflect monitoring of response conflict^19^ and inhibition^12^, did not show differences between inhibition types.

The scalp distribution of the P3 is an important aspect in its functional interpretation. The amplitude often changes over the midline electrodes, increasing in magnitude from frontal to parietal electrode sites^48^, which we also observe in the current study. For the *frontocentral* P3, there was a (non-significant) tendency for an interaction of accuracy and inhibition type. This pattern was probably driven by the higher accuracy difference in the motor inhibition condition. Contrary to traditional binary response tasks, our task contains a variety of possible stimulus–response combinations, increasing the task requirements substantially. These differences in task complexity might prohibit the accuracy effect in the motor inhibition condition from reaching statistical significance. Still, the descriptively higher frontocentral P3 for motor inhibition errors compared to successful motor inhibition might suggest that inhibitory conflict was especially high on error trials and was not suppressed sufficiently^11^. For the *parietal* P3, the interaction of accuracy and inhibition type was significant, and was driven by the substantially lower P3 for successful motor inhibition. At parietal sites, this component is associated with processes such as context updating and attentional resource allocation^15,49^. The successful implementation of motor inhibition and response reselection might require less attentional resources towards context updating and evaluation of the inhibitory outcome than unsuccessful motor inhibition. This interpretation fits the assumed central role of an early inhibitory mechanism in the motor inhibition condition because, when it fails, it implies more resources for the evaluation of the inhibition outcome during later processing. In contrast, an early inhibitory mechanism is not useful in the interference suppression condition, where the stimuli need to be processed thoroughly to resolve conflict on the stimulus level and during response selection. The high resemblance between targets and distractors presumably makes conflict monitoring and evaluation of the inhibition outcome equally resourceful for both errors and correct responses. This is also supported by the longer P3 latencies for responses from the interference suppression condition, which might indicate a longer stimulus evaluation process^15^, as well as the higher number of multiple responses and response corrections compared to the motor inhibition condition.

### Motor execution versus response selection

The response force was substantially lower for motor inhibition errors compared to successful motor inhibition. Studies have shown that responses are less forceful for expected stimuli^50^, suggesting that motor inhibition errors might arise from a lowered motor threshold towards target location, towards which a motor readiness has been adjusted^51^. Additionally, a lowered response force is assumed to reflect online inhibition^52^, suggesting that inhibition was partially implemented but insufficient on trials that resulted in motor inhibition errors.

The stimulus- and response-locked LRP patterns show distinct curves with clearly identifiable onsets for interference suppression errors and successful responses from both inhibition types (Fig. 4), thus representing valid measures for this new task. Surprisingly, for motor inhibition errors no typical LRP curve is shown and the estimated onset of the stimulus-locked LRP lies after the mean response onset. The reason for the lack of a clear LRP signal seems to be conceptual rather than methodological as the other three conditions displayed distinct LRP curves. Indeed, the LRP of motor inhibition errors is marked by multiple small LRP-like curves in the averaged signal, where a clear single onset is difficult to identify. These multiple small deflections indicate response preparations for the opposite response side, which are interrupted by (unsuccessful) attempts of motor inhibition. The LRP results for motor inhibition errors further support an *early* failure of inhibition and a prematurely initiated motor execution process (see also P2). The keypresses for motor inhibition errors were less forceful than for interference suppression errors, suggesting a lowered motor threshold and partial inhibition in this condition. Interference suppression errors, on the contrary, seem to arise from an insufficient response selection process, reflected by the earlier stimulus-locked LRP onset for interference suppression errors compared to successful interference suppression, while motor execution seems unimpeded. By showing that interference suppression is associated with response selection and motor inhibition with response execution, our findings support previous studies^34–36^.

This pattern is supported by the response times, which are faster for motor inhibition errors, underlining that responses were guided by prepotent response tendencies (towards target location) without a proper response selection process. The longer response times for successful responses suggest that a proper response selection process occurred. Responses were slowest for interference suppression errors, indicating that the complex response selection process was impeded and might have been prolonged by motor conflict. For all response types, a substantial decrease in response times can be observed after the first practice block, followed by a saturation for the subsequent blocks (the block-wise response times can be found in the Supplementary Information). This observation can serve as first evidence that participants quickly adapt to the task. An important source for efficient adaptation in the 8ART is the formation of stimulus–response mappings, which enable participants to skip an extensive visual scanning process, which in turn helps them to meet the response time criterion. After the experiment, participants usually report that they tried to memorize the assignment of the stimuli to their fingers to respond faster. This potential formation of S-R-mappings can be validated more systematically in future studies.

### Error processing

After response execution, both early and later error processing (N_e_/P_e_) resembled common literature findings and showed the expected accuracy effects^17,18^. The two types of inhibition errors did not differ significantly regarding the N_e_ amplitude, which is assumed to reflect early conflict-related error monitoring processes^19^. This is in line with results from a previous study by Riesel et al.^3^, who found a reasonably high correlation of the N_e_ across the Flanker task and the Go/NoGo task (*r* = 0.43), and an even higher correlation for the N_e/c_ difference (ΔN_e_, *r* = 0.65). However, they also report a marginally larger ΔN_e_ for the Flanker task compared to the Go/NoGo task, which they interpret as an effect of task difficulty. In our study, there is no evidence for an effect of task difficulty and the two inhibition conditions are more similar regarding physical parameters, which might explain why we do not observe a significant N_e_ difference. In a series of studies, Maier and colleagues found a more pronounced N_e_ for interference suppression errors (there: flanker errors) compared to errors that have other causes such as speed pressure or response confusion (there: non-flanker errors), which they interpret as an indicator of higher error significance^53,54^. They argue that interference suppression errors have a higher error significance because they violate two task goals—responding accurately and suppressing the influence of distractors—whereas other errors only violate the first goal^53^. Following their argumentation, motor inhibition errors might also violate two task goals—responding accurately and suppressing a prepotent response tendency. Hence, the two error types might have a similar error significance, further explaining the absence of significant differences in N_e_ magnitude.

For the P_e/c_, we found a significantly higher amplitude in the motor inhibition condition compared to the interference suppression condition, and a (non-significant) tendency for a higher P_e_ amplitude for motor inhibition errors than for interference suppression errors. A failure in motor inhibition might be more evident than a failure in interference suppression, where deficiencies occur during the more complex response selection. The higher number of multiple responses and response corrections for interference suppression errors might additionally hinder error processing for this error type. In this sense, it would be important to further assess error detection, e.g. with a trial-wise self-evaluation after each response^38^, for behavioural evidence that error detection is indeed better for motor inhibition errors. Interestingly, Riesel et al.^3^ did not find significant differences in P_e_ amplitude between a two-choice Go/NoGo task and a two-choice Flanker task. This suggests that the (non-significant) tendency for a smaller P_e_ for interference suppression errors in our study might have been due to the more complex response selection, an effect that does not seem to unfold in tasks with only two response alternatives.

### Post-response behaviour

Slowing after errors can be interpreted as an adaptive mechanism to avoid subsequent errors due to premature responding^22^, as well as an orienting response towards an expectancy violating event^55^. Our results show slowing after both error types, and less slowing after motor inhibition errors compared to interference suppression errors. For motor inhibition errors slowing might be less useful as an adaptive mechanism. It is in the nature of the task that a motor inhibition error is always followed by a congruent trial (a response guided by response location), thus, slowing to avoid another error might not be necessary. It is more likely that the slowing after motor inhibition errors reflects an orienting response towards the committed error. For motor inhibition errors, the source of the error (i.e. a premature response towards target location) seems easier to identify immediately after the response (i.e. in the time window of the P_e_) than for interference suppression errors (ongoing response conflict), which might be why motor inhibition errors elicit a shorter orienting response compared to interference suppression errors during the following trial. The observed higher pre-error speeding for motor inhibition errors suggests that the sequence of congruent trials preceding motor inhibition errors lowered the motor threshold, and/or increased the speed of evidence accumulation in favour of the target’s location.

## Limitations

With the modified 8ART, we enabled a structured, direct comparison between motor inhibition errors and interference suppression errors. Despite the many similarities, the two inhibition conditions differed in some respects. First, the probability of a need for inhibition was different between conditions. While inhibition was potentially needed on every trial of the interference suppression condition (because distractors were always present), inhibition was only needed on every third to fifth trial in the motor inhibition condition. The resulting differences in cognitive control strategies might cause differences in the reliability of the N_e_ measurement^56^. More precisely, Meyer et al.^56^ found the N_e_ amplitude to be more affected by the number of error trials in a Go/NoGo task (12 error trials required for a correlation of *r* = 0.80 between the N_e_ measured in a subset of error trials and the grand average N_e_) than in a Flanker task (8 trials required). This becomes relevant when the number of error trials included in the ERP analyses falls below the number that is required for sufficient reliability^56^. In our study, after artefact rejection, all 30 datasets contained more than 8 interference suppression errors and 28 datasets contained more than 12 motor inhibition errors (2 datasets contained 11 and 9). Thus, we conclude that the different probabilities of the need for inhibition in the two inhibition conditions do not diminish the reliability of our ERP measures and we consider the differences in inhibition probability essential to evoke the mechanisms of interest.

Next, in the motor inhibition condition participants were confronted with congruent trials (match between stimulus location and response location) and incongruent trials (mismatch between stimulus location and response location), while there were only incongruent trials in the interference suppression condition. We did not include congruent trials in this condition as they induce a different type of interference suppression and might be thus less likely to generate interference suppression errors.

Importantly, to systematically investigate variations in processing from perception to post-response adaptation of two inhibition errors with the new paradigm, we had to make multiple comparisons. Although each performed test targets a different research question, we cannot ensure that they are entirely independent, which would impact the false discovery rate. Consequently, while the alpha level for each individual test amounts to five percent, the global Type-I error rate, i.e. the probability that (at least) one of the effects is a false positive, would be considerably larger. It is important to underline that our investigations were exploratory: we implemented a newly developed complex response task, created two settings tailored to investigate inhibition errors and did not derive a priori assumptions regarding differences between the two error types. Therefore, while our study delivered important findings and serves as a solid basis for further investigations, further research is needed to replicate our findings (in particular regarding interactions of accuracy and inhibition type for the P2 and P_e_, which only surfaced as non-significant tendencies).

## Conclusion

In our study, we elucidated the neural and behavioural antecedences and consequences of motor inhibition errors and interference suppression errors in a complex choice task. Our findings suggest that motor inhibition errors arise from a deficient early inhibitory process at the levels of perception and motor execution that precedes the start of a more complex response selection process. Early attention-related processing mechanisms seem intact for motor inhibition errors. On the bridge to later, more elaborate processing, information that cues the prepotent response tendency might not be suppressed but processed further, and conflict evaluation becomes more resourceful. In the end, this early failure of perceptual and motor inhibition might lead to a more pronounced error evidence accumulation process. In contrast, our findings indicate that interference suppression errors arise during the complex response selection process. Along the entire processing stream, they appear more similar to their correct counterparts. Interference suppression errors are accompanied by a high number of multiple responses and response corrections, which together with the more complex response selection process presumably leads to less error evidence accumulation. Naturally, replication studies are needed and the relationship between the two error types can be investigated further, for example, by addressing error detection.

## Method

### Participants

We collected data from 41 participants. We had to exclude datasets from several participants due to technical issues (defect light sensor: *n* = 6), insufficient data quality (not enough trials left after artefact rejection: *n* = 3) and insufficient number of trials of at least one response type (at least six trials necessary for error-related ERP components: *n* = 2; see 41). The final sample comprised 30 undergraduate psychology students (9 males, 19 females, 2 diverse; age: *M* = 23.9 years, *SD* = 6.0 years). This sample size allowed uncovering effect sizes of η_p_^2^ ≥ 0.22 including interactions, given a Type I error of 0.05 and a power of 0.80 (MorePower;^57^). We used an online system^58^ for participant recruitment. The participants were rewarded with course credits and reported normal or corrected-to-normal vision. The ethical board of the Faculty of Humanities at the University of Cologne approved the study and written consent was collected from each participant.

### Procedure

**Figure 6:**
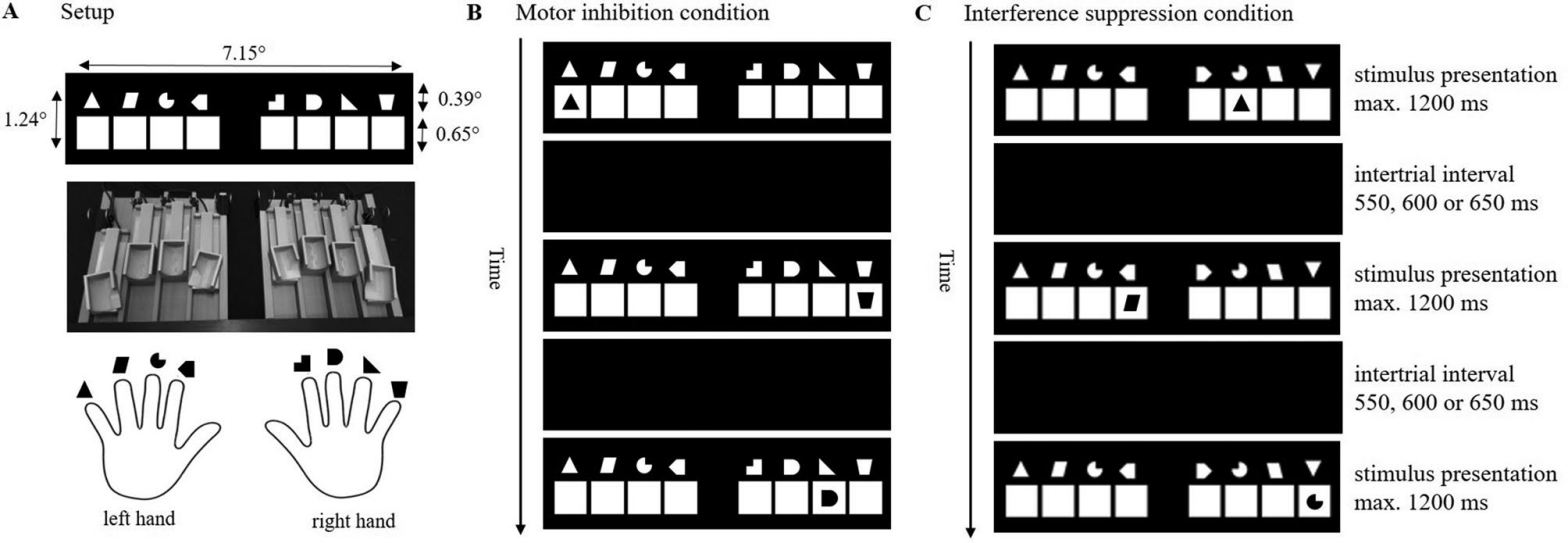
Setup of experiment and task design. *Note* (**A**) Setup of the experiment: stimulus presentation including visual angles, force-sensitive response keys and stimulus*–*response assignment; (**B**) exemplary trials from the motor inhibition condition: the first two trials induce a prepotent response tendency (location matches stimulus identity) and in the third trial the location indicates an incorrect response tendency that has to be inhibited; (**C**) exemplary trials from the interference suppression condition with a target stimulus and a mirrored distractor stimulus.

We implemented a modified version of the 8ART^37,38^, where participants responded to appearing stimuli according to an instructed stimulus–response assignment in a given response time window (Fig. 6). The task consisted of several trials, in each of which eight white squares were presented in the centre of a black screen. A different shape was located above each square. The participants placed their fingers, excluding their thumbs, on eight force-sensitive response keys. The response keys were assigned to the shapes above the squares. During each trial, one of the shapes also appeared in one of the eight white squares. The participants were instructed to respond to the appearing shape with the finger it was assigned to as fast and as accurately as possible. For this, they were given a response time limit of 1000 ms. As soon as the participants responded with one of the eight response keys, the trial was finished, and the next trial started. The intertrial interval randomly varied between 550, 600 and 650 ms. If a response exceeded the response time limit, the feedback ‘even faster’ (German: *noch schneller*) appeared on the screen. No other feedback was given to the participants.

The experiment consisted of two within-subject conditions. The first condition aimed at evoking prepotent response tendencies (*motor inhibition condition*). In this condition, the shapes appeared more frequently in the white square beneath their matching shape (congruent trials) with the purpose of creating an association between response and stimulus location. Every three to five trials, the appearing shape was not presented beneath its matching shape (incongruent trials). A retrieval of previously encoded associations between response and stimulus location would lead to an erroneous response. Instead, participants had to ignore this association and respond to the stimulus identity instead. Thus, motor inhibition is required when the more frequent stimulus–response assignments create prepotent response tendencies that need to be overridden on the less frequent trials (exemplary trials are depicted in Fig. 6B). The condition consisted of 10 blocks with 80 trials each, leading to a total of 800 trials for this condition. Short breaks between blocks ensured time to rest for the participants.

The second condition aimed at evoking responses based on distractor stimuli (*interference suppression condition*). This condition included the four shapes of the left side from the motor inhibition condition which were mirrored regarding their spatial orientation, leading to a set of eight shapes that resembled each other, serving as the interference-inducing distractor stimuli. We always mirrored the same four shapes to avoid differential effects of shifting or re-learning between the conditions. The appearance of the shapes inside the white squares varied randomly in location, with all shape–location combinations having the same probability. However, the shapes never appeared beneath their matching shape (as this would decrease the need for interference suppression) or their individual counterpart (as this would permit differentiating responses guided by distractor influence from responses guided by stimulus location). Interference suppression is required when the distractors induce conflict (e.g. regarding stimulus processing or response selection), which needs to be resolved in order to respond correctly (exemplary trials are depicted in Fig. 6C). This condition consisted of six blocks with 80 trials each, leading to a total of 480 trials for this condition. In total, the motor inhibition condition contained more trials than the interference suppression condition because the congruent trials were used to generate prepotent response tendencies but were not of interest for the analyses as no inhibition was required.

Each inhibition condition was split into two parts and the order of conditions was balanced between participants (ABAB/BABA design) to avoid sequence effects. Each part started with two short practicing blocks consisting of 20 trials where participants were given the opportunity to get used to the task set and the response time limit. The total duration of the experiment was approximately 45 min, including breaks.

### Apparatus

Figure 6A shows the set of stimuli and their visual angles. The physical centre of the screen is marked by the black square in the middle between the two rows of four white squares. A chin rest was used to constrain excessive head movements and to keep the screen distance (88 cm) constant. A luminance-sensitive photosensor was mounted on the bottom corner of the screen to assess real-time stimulus onsets. The eight response keys measured response force via embedded force sensors (FCC221-0010-L, DigiKey MSP6948-ND). A VarioLab AD converter digitized the response signal at a sampling rate of 1024 Hz. When the sensors registered the exceedance of 40 cN on one of the response keys, the keypress was registered as a response. The response keys were calibrated to the weight of the participant’s fingers and adjusted to their length.

### Electrophysiological data

The electroencephalogram (EEG) signal was recorded with 63 active Ag/AgCl electrodes (Acticap, Brain Products) that were placed onto the scalp according to the standard international 10–20 system^59^. An electrode placed on the left mastoid served as an online reference. The averaged signal from the left and right mastoids was used for offline re-referencing. The electrooculogram (EOG) was recorded with four passive bipolar Ag/AgCl electrodes (ExG-Amplifier, Brain Products) positioned horizontally next to the eyes and vertically above and below the left eye. A BrainAmp DC amplifier (Brain Products) recorded the EEG and EOG signals continuously at a sampling rate of 500 Hz and a filter from DC to 70 Hz.

We applied a 50 Hz notch filter to clean the data from line noise. For the stimulus-locked ERP components, we locked the trial intervals onto the stimulus onset and segmented time windows from − 100 to 700 ms. For the response-locked components, we locked the trial intervals onto the response onset and segmented time windows from − 100 to 500 ms. We performed baseline corrections based on the 100 ms before stimulus onset or response onset, respectively. We corrected for eye blinks with an ocular correction algorithm^60^ and rejected artefacts where ERP waves exceeded ± 100 μV. To clean each electrode’s activity from the activity of nearby electrodes and to detach the signal from the reference electrode we carried out a current source density (CSD) analysis^61^.

The assessment specifics of the investigated ERP components are listed in Table 3. The time windows and electrode sites for the ERP components were determined based on literature (P1, N1:^4,62^; P2:^7^, N2, P3:^63^; N_e/c_, P_e/c_:^64^) and validated through visual inspection. We quantified the mean amplitudes as ± 2 data points around the peak on the participant level separately for each condition. The averaged activity from five data points decreases the influence of noise opposed to a simple peak measure^65^ and the narrow time window for the sharp components with distinct peaks avoided covering activity from preceding and subsequent components. For the slower components (P3, P_e/c_) we additionally averaged activity from 40 data points (see Supplementary Information).

**Table 3:** Information on the assessed event-related potential (ERP) components.

Component	Time window	Electrode	Quantification	Event	CSD
P1	90–160 ms	PO7, PO8	Mean amplitude	Stimulus-locked	Yes
N1	150–210 ms	P1, P2	Mean amplitude	Stimulus-locked	Yes
P2	150–250 ms	PO9, PO10	Mean amplitude	Stimulus-locked	Yes
N2	150–300 ms	FCz	Mean amplitude	Stimulus-locked	Yes
P3	200–500 ms	FCz, Pz	Mean amplitude	Stimulus-locked	No
N_e/c_	0–150 ms	FCz	Mean amplitude	Response-locked	Yes
P_e/c_	150–300 ms	Cz	Mean amplitude	Response-locked	Yes
LRP	− 100–800 ms	C3´, C4´	LRP onset	Stimulus-locked	No
LRP	− 500–200 ms	C3´, C4´	LRP onset	Response-locked	No

For the P1, the N1 and the P2, where the signal was not distributed centrally, we averaged activity from both electrode sites where the signal was largest. Mean amplitude is defined as ± 2 data points around the peak. When indicated, we conducted CSD analyses to obtain a signal that is cleared from activities of neighbouring electrodes^70^. For the P3, we report results of the untransformed data as visual inspection showed that it was quite distorted by the CSD analyses. This can be explained by the fact that the P3 emerges from activity across the entire midline of the scalp (for topographies see Results), where it is interpreted in different ways depending on where it is maximal (for details see Discussion). LRP refers to the lateralised readiness potential.

The LRP is defined as the average of the two differences [C4′(*t*) − C3′ (*t*)] for the left hand and [C3′(*t*) − C4′(*t*)] for the right hand^8^, using the jack-knifing method to estimate LRP onsets^16,66,67^ and transforming the jack-knifed onset values according to Smulders^68^. While the duration of stimulus processing and response selection can be assessed by examining the time from stimulus onset to LRP onset, the duration of response activation and motor processes can be assessed by examining the time from LRP onset to response onset^69^. We used a 50% criterion to determine the LRP onset for both the stimulus-locked and the response-locked LRP (i.e. the onset was determined at the first time point where the LRP exceeded 50% of the peak amplitude for at least five consecutive time points). To enable comparisons between correct responses and errors, we based LRP computations for errors on the hands with which the responses were executed instead of the hands with which the correct response would have been executed. For the LRP data we applied a 30 Hz low-pass filter at the end of the processing pipeline.

### Statistical analyses

**Figure 7:**
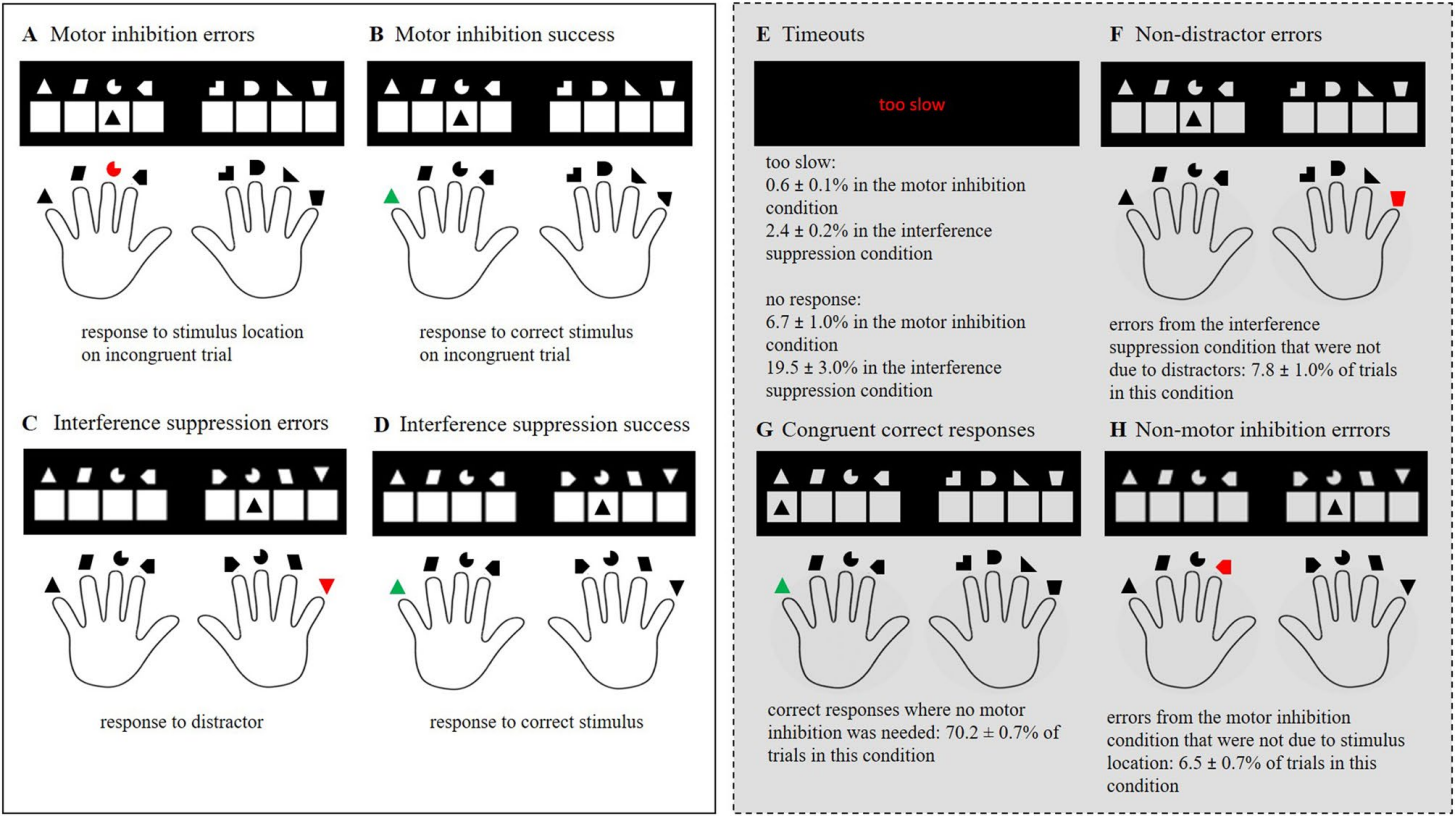
Response types and excluded trials. *Note* The left panel shows examples for (**A**) a motor inhibition error, (**B**) motor inhibition success, (**C**) an interference suppression error, and (**D**) interference suppression success. The right panel shows examples for excluded trials that are (**E**) too slow responses and trials where no response was given, (**F**) errors from the interference suppression condition that were not responses to the distractor, (**G**) correct responses from the motor inhibition condition on congruent trials, and (**H**) errors from incongruent trials of the motor inhibition condition that where not responses to stimulus location.

We included four response types in our analyses: motor inhibition errors, motor inhibition success, interference suppression errors, and interference suppression success. Figure 7 gives an overview over the four response types and the excluded trials. A comparison of the excluded congruent trials from the motor inhibition condition with the incongruent trials (i.e. motor inhibition success and errors) can be found in the Supplementary Information.

We analysed median RT, the percentage of multiple responses and the peak force with a series of 2-by-2 repeated-measures ANOVAs with the factors *inhibition type* (motor inhibition vs. interference suppression) and *accuracy* (success vs. error). Post hoc tests (Tukey’s HSD for within-comparisons) were implemented for significant ANOVA interactions. RT was defined as the time interval from stimulus onset to the exceedance of a force of 40 cN on one of the response keys. Multiple responses were defined as trials where the response force exceeded 40 cN on more than one key. We contrasted the percentage of response corrections of motor inhibition errors and interference suppression errors with a two-tailed *t*-test. We computed PES by subtracting the averaged RT of trials preceded by motor inhibition/interference suppression success from the averaged RT of trials preceded by motor inhibition/interference suppression errors. As the visual inspection of our data did not indicate a confound of error frequencies and response time level, there was no need to quantify PES as a difference measure of pre-error trials and post-error trials (^71^; for details on the appropriateness of PES quantifications see^72^). Additionally, based on the recommendations of Pfister and Foerster^72^ we computed pre-error speeding with the same method to assess whether motor inhibition errors are preceded by more speeding (than interference suppression errors) as the preceding series of congruent trials should lower the motor threshold in this condition. We analysed whether motor inhibition errors and interference suppression errors showed PES and pre-error speeding that differed significantly from zero (one-sided *t*-tests) and whether the two error types differed from of each other regarding PES and pre-error speeding (two-sided *t*-tests). We contrasted the post-response improvement of accuracy between motor inhibition errors and motor inhibition success and between interference suppression errors and interference suppression success (two-tailed *t*-tests). We did not compare the post-response improvement of accuracy between inhibition types because in the motor inhibition condition the probability of a subsequent correct response was inherently higher. We analysed all ERP parameters with a series of 2-by-2 repeated-measures ANOVAs with the factors *inhibition type* (motor inhibition vs. interference suppression) and *accuracy* (success vs. error). Post hoc tests (Tukey’s HSD for within-comparisons) were implemented for significant ANOVA interactions.

Depicting cognitive processes from stimulus presentation to post-response adaptation implies conducting a series of inference statistical tests. One possibility to deal with this multitude of tests is to adjust the alpha level, ensuring a global Type-I error rate of 5% for the entire study. While the issue of multiple comparison conduction is certainly of importance (for ERP research see^73^), such a strict way of testing conflicts with the exploratory purpose of this study. As the main goal was to generate first insights into the differences between motor inhibition errors and interference suppression errors, we report the uncorrected statistics in the following, but naturally replication studies are needed.

### Ethical approval

Approval was obtained from the ethics committee of the Faculty of Human Sciences at the University of Cologne. The procedures used in this study adhere to the tenets of the Declaration of Helsinki.

### Consent to participate

Informed consent was obtained from all individual participants included in the study.

### Supplementary Information

Supplementary Information.

## Data Availability

The aggregated data and analysis scripts are available at OSF under the following link [https://osf.io/r4yeq/?view_only=cd628b937e7d410a9c349399afa3c4c7]. The study was not preregistered.
