# Supplementary material for: Motor inhibition errors and interference suppression errors differ systematically on neural and behavioural features of response monitoring
Source: Sci Rep. 2024 Jul 10;14:15966. doi: 10.1038/s41598-024-66364-8 (PMC11237018; doi:10.1038/s41598-024-66364-8)
Supplement: Supplementary file 1 — Supplementary Information. [file 41598_2024_66364_MOESM1_ESM.pdf]

Motor inhibition errors and interference suppression errors differ systematically on neural and behavioural features of response monitoring

Elisa Porth, André Mattes, Jutta Stahl

Department of Individual Differences and Psychological Assessment

University of Cologne

Cologne, Germany

Supplementary Information

Supplementary Information

Topographies of the P3

Figure 1

Topographical maps around the time window of the P3

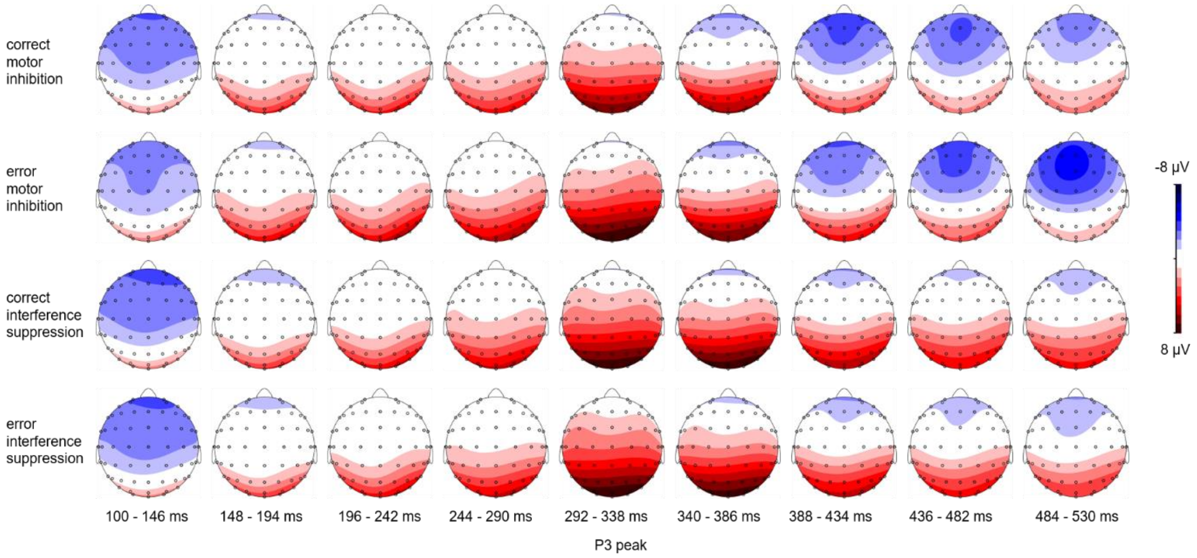

Note. Averaged topographical maps separately for inhibition type (motor inhibition, interference suppression) and accuracy (correct, error) in the time window of 100–530 ms after stimulus onset.

Paired Differences of the ERP Waveforms

Figure 2

Paired differences of the stimulus-locked ERP waveforms P2, N2 and P3

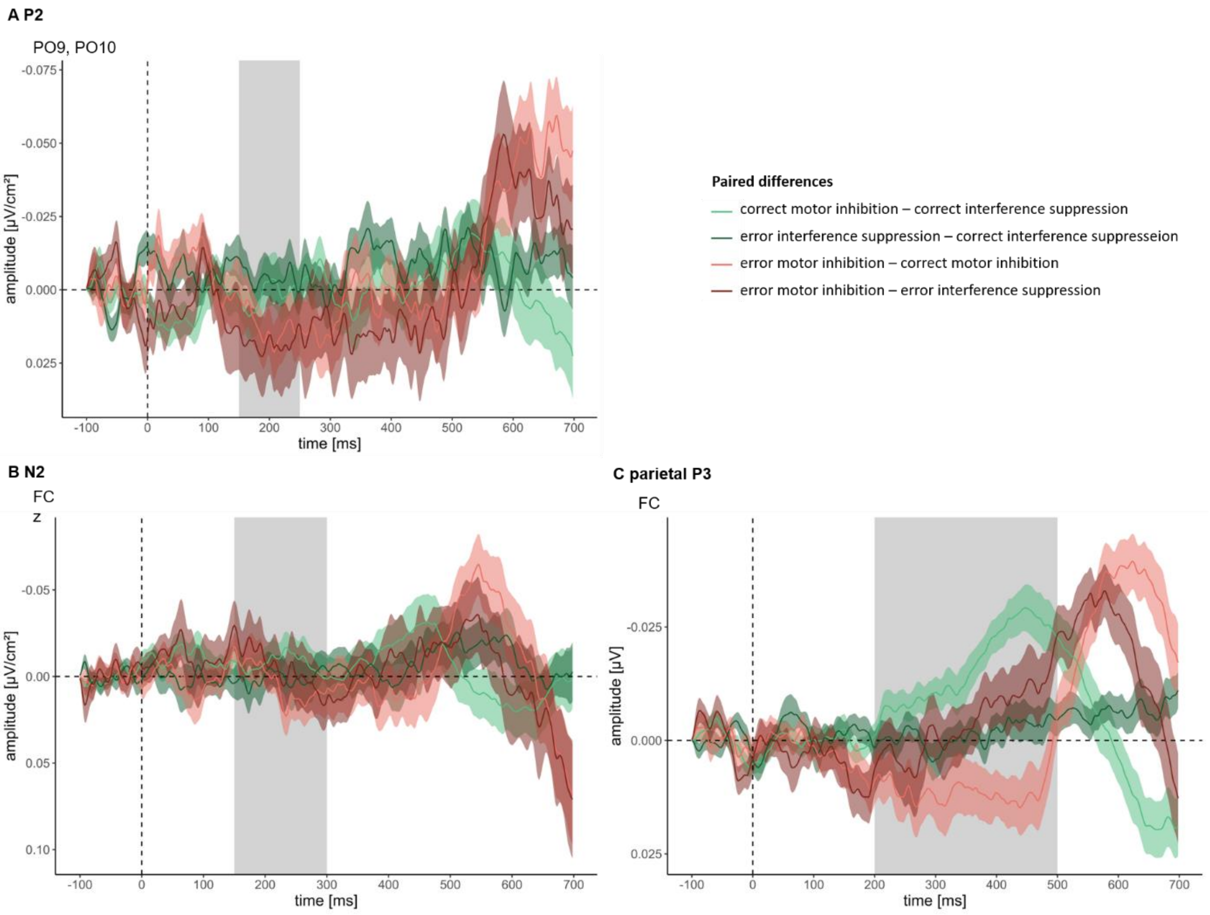

Note. Averaged stimulus-locked waveforms for paired-differences between inhibition types (motor inhibition, interference suppression) and accuracies (correct, error) (A) of the current-source density transformed P2, measured at PO9 and PO10 in the time window of 150–250 ms after stimulus onset; (B) of the current-source density transformed N2, measured at FCz in the time window of 150–300 ms after stimulus onset; (C) of the parietal P3, measured at Pz in the time window of 200–500 ms after stimulus onset.

Figure 3

Paired differences of the response-locked ERP waveforms $N_{e/c}$ and $P_{e/c}$

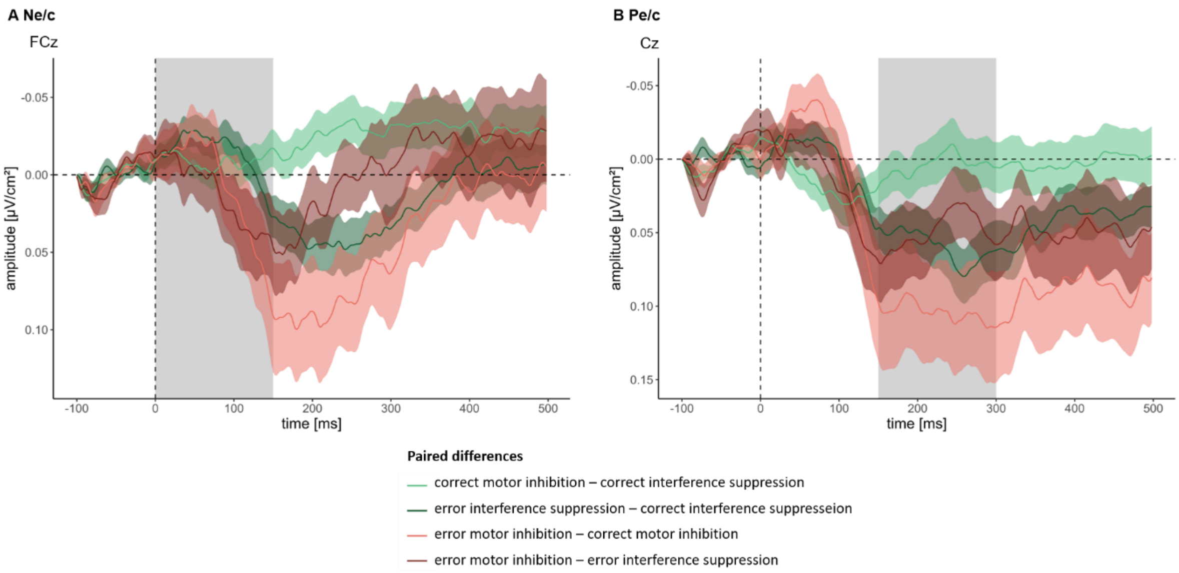

Note. Averaged current-source density transformed waveforms for paired-differences between inhibition types (motor inhibition, interference suppression) and accuracies (correct, error) (A) of the error negativity, measured at the FCz in the time window of 0–150 ms after response onset; (B) of the error positivity, measured at the Cz in the time window of 150–300 ms after response onset.

Paired Differences of the LRP Onsets

Figure 4

Paired differences of the stimulus-locked and response-locked LRP onsets

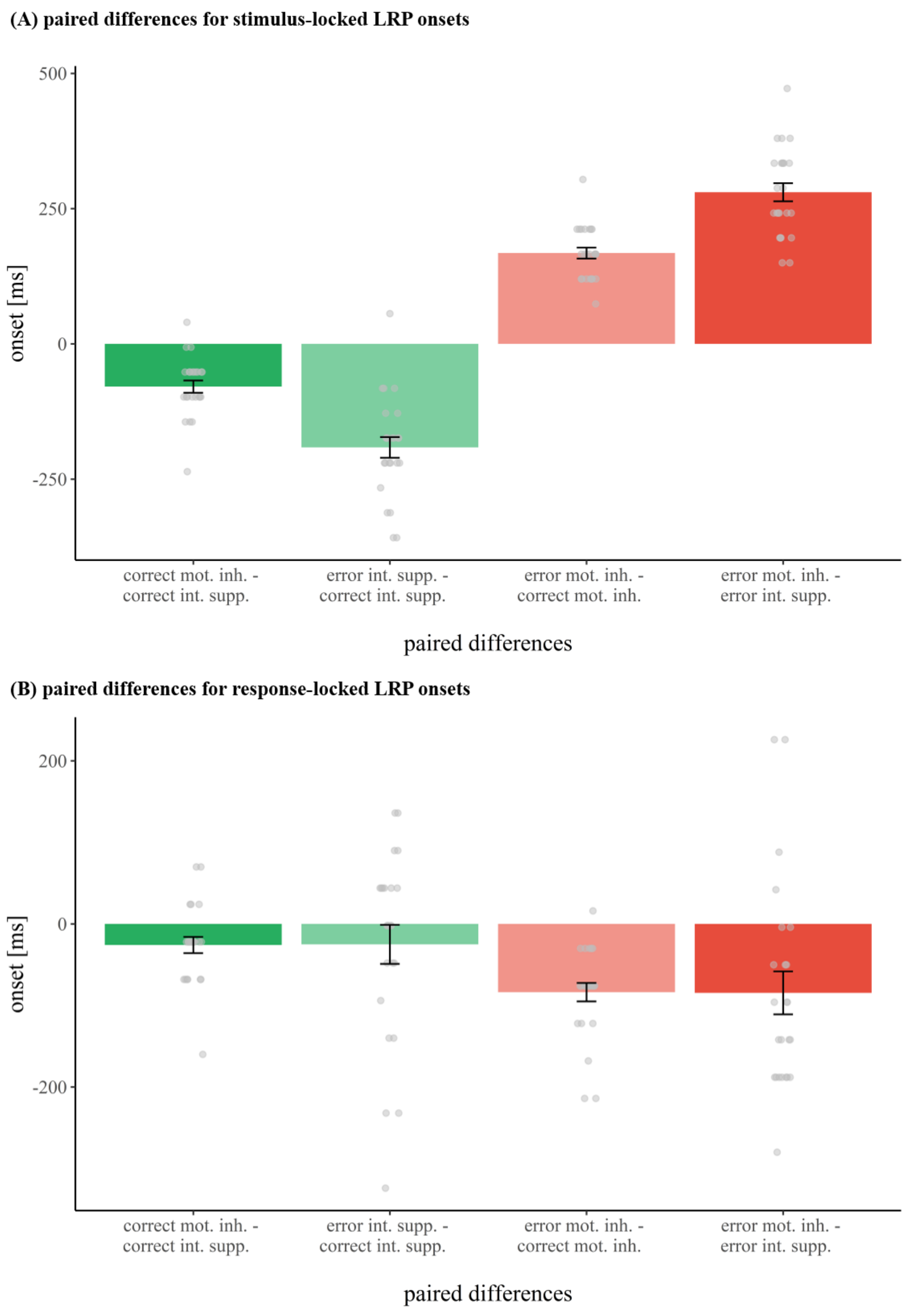

Note. Paired differences of the onsets of the lateralised readiness potential between inhibition types (motor inhibition, interference suppression) and accuracies (correct, error) (A) for the stimulus-locked onsets; (B) for the response-locked onsets.

Block-wise response times for each response type

Figure 5

Response times separately for each block and each response type

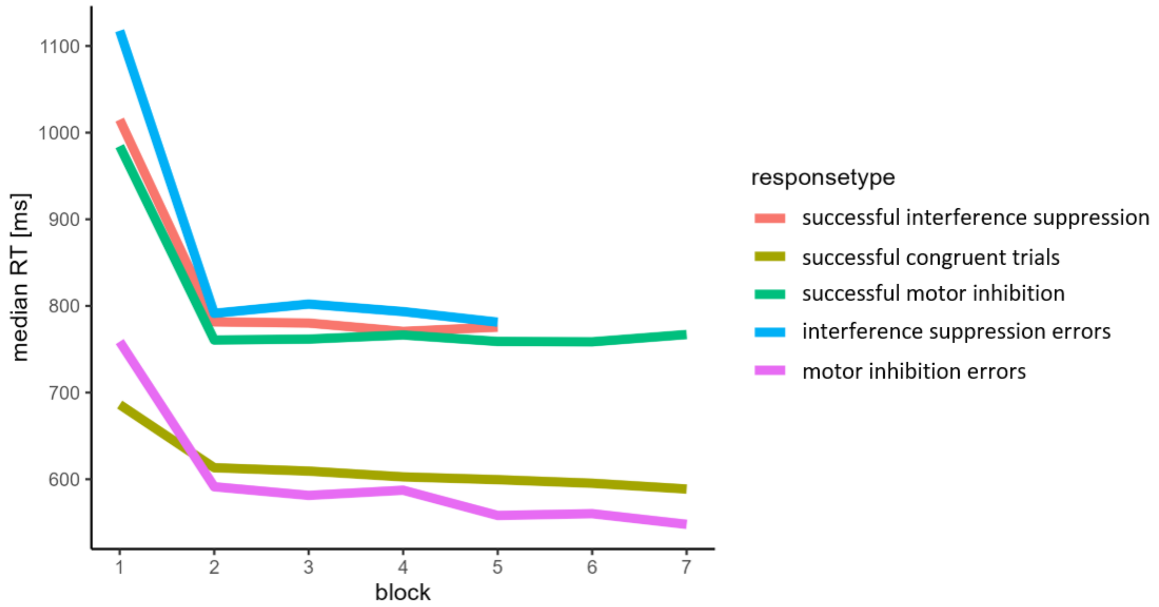

Note. Block-wise median response times separately for inhibition type (motor inhibition, interference suppression) and accuracy (correct, error).

P3 mean activity

For the P3 mean activity (motor inhibition success: 1.2 ± 0.7 µV; motor inhibition errors: 1.8 ± 0.7 µV; interference suppression success: 2.1 ± 0.6 µV; interference suppression errors: 2.0 ± 0.7 µV), there was no significant main effect of Accuracy, $F(1, 29) = 0.50$, $p = .484$, $\eta_p^2 = .02$, no significant main effect of Inhibition Type, $F(1, 29) = 2.53$, $p = .123$, $\eta_p^2 = .08$, and no significant interaction of Accuracy and Inhibition Type, $F(1, 29) = 2.98$, $p = .100$, $\eta_p^2 = .09$.

$P_{e/c}$ mean activity

For the $P_{e/c}$ mean activity, there was a significant main effect of Accuracy, $F(1, 29) = 12.21$, $p = .002$, $\eta_p^2 = .30$, and Inhibition Type, $F(1, 29) = 8.40$, $p = .007$, $\eta_p^2 = .22$, and a non-significant tendency for an interaction of Accuracy and Inhibition Type, $F(1, 29) = 3.94$, $p = .057$, $\eta_p^2 = .12$. Although this effect does not reach a 5% significance level, we cannot

rule out that it was driven by a larger $P_{e/c}$ mean activity for motor inhibition errors (0.14 ± 0.03 μV/cm^2) than for the other three response types (motor inhibition success: 0.04 ± 0.02 μV/cm^2; interference suppression success: 0.03 ± 0.02 μV/cm^2; interference suppression errors: 0.08 ± 0.02 μV/cm^2), and a larger mean activity for interference suppression errors than for interference suppression success.

Behavioural Data for Successful Congruent Trials of the Motor Inhibition Condition

The response times for successful congruent trials (i.e. trials at which no inhibition was needed) were significantly faster (M = 581.8 ms, SE = 10.6 ms) than for successful incongruent trials (M = 754.6 ms, SE = 11.2 ms), $t(29) = 23.72$, $p <.001$, $d = 4.33$, and significantly slower than for motor inhibition errors (M = 538.7 ms, SE = 9.5 ms), $t(29) = 7.13$, $p <.001$, $d = 1.30$.

The response force for successful congruent trials was significantly lower (M = 141.5 cN, SE = 10.6 cN) than for successful incongruent trials (M = 189.8 cN, SE = 15.6 cN), $t(29) = 7.05$, $p <.001$, $d = 1.29$, and did not differ significantly from motor inhibition errors (M = 155.6 cN, SE = 11.5 cN), $t(29) = 1.94$, $p =.062$, $d = 0.35$.

Motor inhibition errors were always preceded and followed by at least two congruent trials. Figure 6 shows the response times of the peri-error correct congruent trials for motor inhibition errors (blue line) and the mean response times for successful congruent and incongruent trials. For comparison, the same information is displayed for the interference suppression condition (red line). Motor inhibition errors and interference suppression errors are both marked by substantial pre-error speeding [1]. On the error trial itself, however, motor inhibition errors maintain short response times, comparable in size to the response times of the preceding correct trials. Interference suppression errors, on the contrary, display a substantial increase in response times.

Figure 6

Response times of the peri-error trials for motor inhibition errors and interference suppression errors

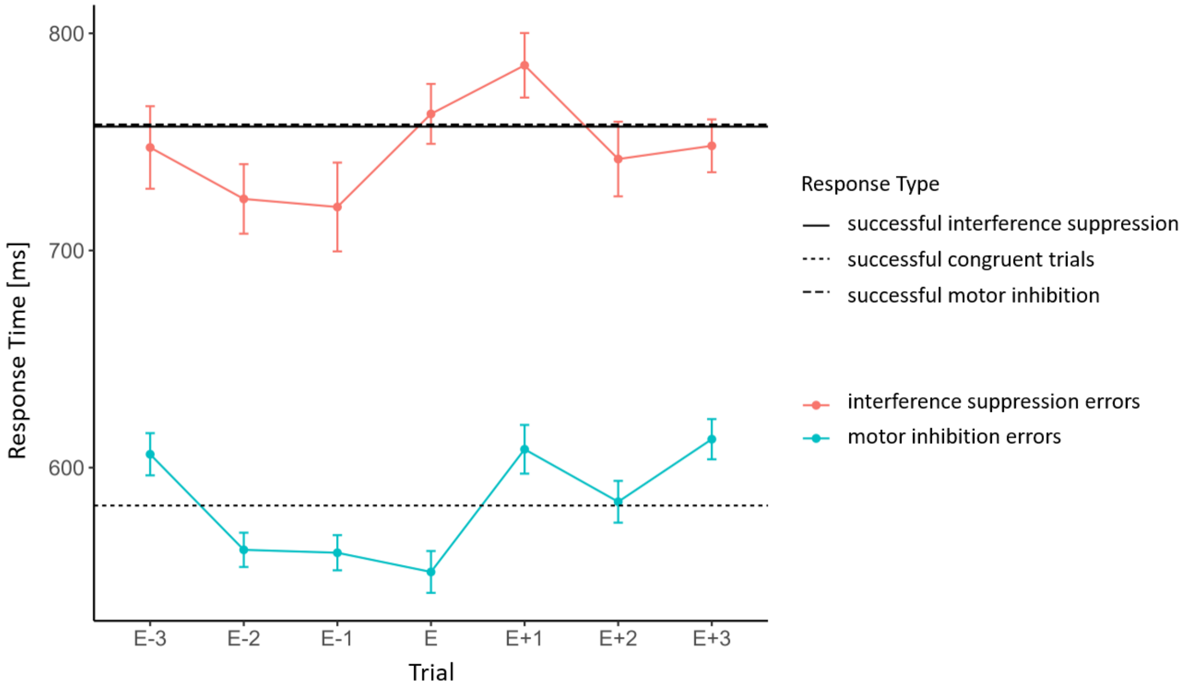

Note. Response times of the peri-error correct congruent trials for motor inhibition errors (blue line), interference suppression errors (red line), and the mean response times for successful congruent and incongruent trials from the motor inhibition condition and successful interference suppression (straight lines).

The pre-error speeding and the fast response times for motor inhibition errors together with an above-chance rate of motor inhibition errors (*M* = 26.7 % vs 12.5 % chance level for eight response alternatives) are clear indicators for prepotent response tendencies towards target location. This is further supported by the lower response force for successful congruent trials and motor inhibition errors compared to successful incongruent trials. Studies have shown that responses are less forceful for expected [2] towards which a motor readiness has been adjusted [3].

References

1. Pfister, R. & Foerster, A. How to measure post-error slowing: The case of pre-error speeding. *Behavior Research Methods* **54,** 435–443 (2022).
2. Jaskowski, P. & Verleger, R. A clock paradigm to study the relationship between expectancy and response force. *Perceptual and motor skills* **77,** 163–174 (1993).
3. Mattes, S. & Ulrich, R. Response force is sensitive to the temporal uncertainty of response stimuli. *Perception & Psychophysics* **59,** 1089–1097 (1997).
